# Developing an emergency ultrasound app – a collaborative project between clinicians from different universities

**DOI:** 10.1186/s13049-015-0130-2

**Published:** 2015-06-20

**Authors:** Kim Thestrup Foss, Yousif Subhi, Rasmus Aagaard, Ebbe Lahn Bessmann, Morten Thingemann Bøtker, Ole Graumann, Christian B. Laursen, Jesper Weile, Tobias Todsen

**Affiliations:** Centre for Clinical Education, Centre for HR, Capital Region of Denmark & University of Copenhagen, Copenhagen, Denmark; Department of Neurology, Copenhagen University Hospital Herlev, Herlev, Denmark; Clinical Eye Research Unit, Copenhagen University Hospital Roskilde, Roskilde, Denmark; Research Center for Emergency Medicine, Aarhus University, Aarhus, Denmark; Department of Anesthesia, Randers Regional Hospital, Randers, Denmark; Department of Anesthesia, Copenhagen University Hospital Herlev, Herlev, Denmark; Research Department, Prehospital Emergency Medical Services, Central Denmark Region, Aarhus, Denmark; Center of Clinical Ultrasound, Faculty of Health, Aarhus University, Aarhus, Denmark; Department of Radiology, Aarhus University Hospital, Aarhus, Denmark; Institute of Clinical Research, Aarhus University, Aarhus, Denmark; Institute of Clinical Research, University of Southern Denmark, Odense, Denmark; Department of Respiratory Medicine, Odense University Hospital, Odense, Denmark; Department of Emergency Medicine, Regional Hospital Herning, Herning, Denmark; Greenland Center for Health Research, University of Greenland, Nuuk, Greenland

**Keywords:** Focused ultrasound, Medical education, Medical mobile applications, mHealth, e-learning, Video-assisted learning

## Abstract

Focused emergency ultrasound is rapidly evolving as a clinical skill for bedside examination by physicians at all levels of education. Ultrasound is highly operator-dependent and relevant training is essential to ensure appropriate use. When supplementing hands-on focused ultrasound courses, e-learning can increase the learning effect. We developed an emergency ultrasound app to enable onsite e-learning for trainees. In this paper, we share our experiences in the development of this app and present the final product.

## Letter to the Editor

Focused ultrasound is rapidly expanding in emergency care in both prehospital and hospital settings, but ultrasound is operator-dependent and requires adequate training [1–3]. Ultrasound courses provide the trainees with confidence in clinical use [4] and e-learning is excellent course-preparation [5]. Clinicians frequently use smartphones for educational purposes and an app could potentially provide e-learning support for hands-on training in focused ultrasound [6]. We sought to develop a non-commercial smartphone app for focused ultrasound in emergency settings independent of financial interests. The purpose of this letter is to report on the process developing a clinical teaching app, and to present the final product.

We formed a group of Danish physicians engaged in ultrasound teaching and research. Our goal was to produce a smartphone app with standardized national instructions on how to conduct focused ultrasound in an emergency setting. Engaging physicians from different hospitals, universities, and specialities enabled us to elevate instructions beyond local practice to be nationally applicable. Skype (Skype Technologies, Luxembourg) was used for meetings to discuss the content of the app and gain consensus about nomenclature and probe orientation. We agreed that an introduction to focused ultrasound and five specific focused ultrasound protocols (focused lung ultrasound, focus assessed transthoracic echocardiography, ultrasound guided vascular access, extended focused assessment with sonography for trauma, and limited compression ultrasonography for deep venous thrombosis) would be appropriate. Main authors were selected for each protocol-section and a manuscript was drafted and shared with the other authors using Dropbox (Dropbox Inc., USA) for review. After review by the other authors, the main authors finalized each section. Video-demonstrations were recorded in Clinical Skills Laboratories at Centre for Clinical Education (Copenhagen, Denmark). Videos were recorded with three-point lighting setup and three video cameras installed on tripods with a portable microphone for audio. Ultrasound cine-loops were recorded directly from the ultrasound machines (from GE Healthcare, UK and BK Medical, Denmark) using MediCapture (MediCapture, USA). Videos and cine-loops were edited using Final Cut X (Apple Inc., USA) and uploaded in QuickTime Movie format (.mov) to an online video-hosting service (Vimeo.com, USA) for easy embedding into the app.

**Fig. 1:**
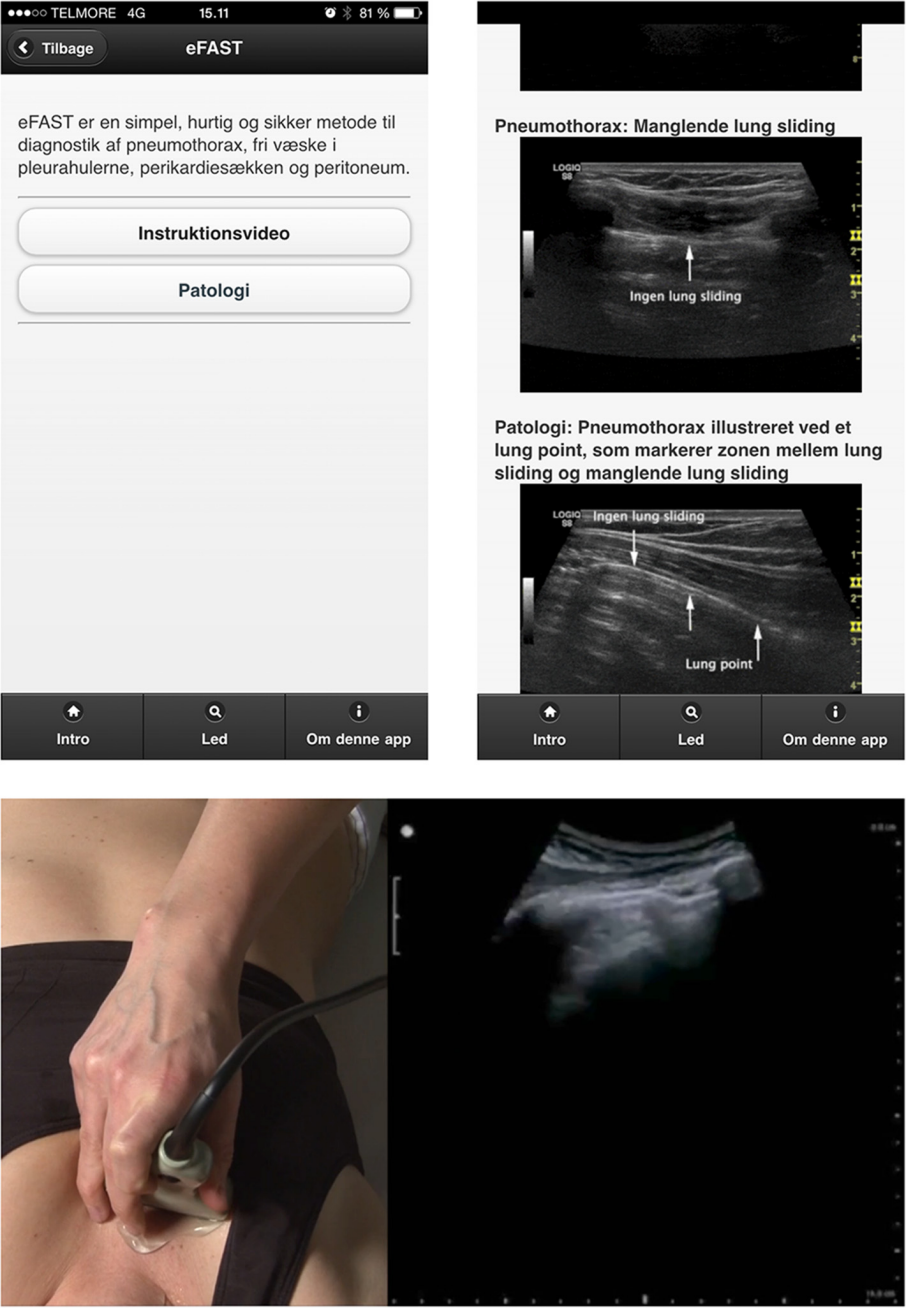
Acute Ultrasound App: Example of app overview of eFAST (top, left) and examples of how to detect pathology (top, right). Example of an instructional video (bottom)

The app was developed using a previously described simple web-app method [7, 8] and the format enabled easy and multi-platform access. Screenshots of the final app are provided in Fig. 1 and the app is accessible from: http://akutul.cekuapp.dk

In conclusion, developing an educational smartphone app on focused ultrasound through nationwide collaboration across university hospitals was feasible.

This app cannot stand alone, nor replace supervision or courses in focused ultrasound, but it is a useful on-site e-learning supplement that may enhance learning outcomes.
